# Optical Full Adder Based on Integrated Diffractive Neural Network

**DOI:** 10.3390/mi16060681

**Published:** 2025-06-04

**Authors:** Chenchen Deng, Yilong Wang, Guangpu Li, Jiyuan Zheng, Yu Liu, Chao Wang, Yuyan Wang, Yuchen Guo, Jingtao Fan, Qingyang Du, Shaoliang Yu

**Affiliations:** 1 Beijing National Research Center for Information Science and Technology, Tsinghua University, Beijing 100084, China; chenchendeng@tsinghua.edu.cn (C.D.); chaow@tsinghua.edu.cn (C.W.); wangyuyan@mail.tsinghua.edu.cn (Y.W.); guo-yuchen@tsinghua.edu.cn (Y.G.); fanjingtao@tsinghua.edu.cn (J.F.); 2 Department of Automation, Tsinghua University, Beijing 100084, China; 3 Zhejiang Lab, Hangzhou 311121, China; qydu@zhejianglab.edu.cn (Q.D.); yusl@zhejianglab.com (S.Y.)

**Keywords:** optical computing, diffractive neural network, logic computing

## Abstract

Light has been intensively investigated as a computing medium due to its high-speed propagation and large operation bandwidth. Since the invention of the first laser in 1960, the development of optical computing technologies has presented both challenges and opportunities. Recent advances in artificial intelligence over the past decade have opened up new horizons for optical computing applications. This study presents an end-to-end truth table direct mapping approach using on-chip deep diffractive neural network (D^2^NN) technology to achieve highly parallel optical logic operations. To enable precise logical operations, we propose an on-chip nonlinear solution leveraging the similarity between the hyperbolic tangent (tanh) function and reverse saturable absorption characteristics of quantum dots. We design and demonstrate a 4-bit on-chip D^2^NN full adder circuit. The simulation results show that the proposed architecture achieves 100% accuracy for 4-bit full adders across the entire dataset.

## 1. Introduction

The growth of computer performance has been driven by the miniaturization of transistors over the past 60 years following Moore’s law. However, recently, semiconductor advancements have slowed down below the pace of Moore’s law as transistor scales approach physical limits, and the further sustainable development of computing processors cannot be supported [1]. It is imperative to explore new computing architectures to enhance computational capacity. Lightwave, which can carry information in multiple dimensions, propagates at the fastest speeds and has been applied in task-specific computing architectures such as image classification. However, light as a computing medium for general purpose computing has met tremendous challenges and is of limited scale. Conventional optical logic operations have followed the roadmap of the electronic logic gates based on Boolean algebra. Basic logic gates [2,3,4] are implemented and combinational logic operations such as half-adders, full-adders [5,6], and optical cross-connects [7] are constructed from basic logic gates. The optical logic operations usually require high-order optical nonlinear devices like semiconductor optical amplifiers (SOAs) [8,9], photonic crystals [6], micro-ring cavities [10], and plasmonic devices [5,11]. These devices were initially designed mainly for long-haul communication regimes. Although a lot of endeavors have been made to reduce the power consumption of optical components, the lowest reported power consumption in logic information generation is 1 pJ/bit [12], which is not yet comparable to the sub-fJ/bit switching energy cost of CMOS gates. If optical logic operations were to emulate their electronic counterparts by cascading single-bit logic gates according to Boolean algebra, the overall energy consumption would escalate further.

With the escalating development of artificial intelligence, another technical approach towards highly energy-efficient and compact optical logic computing is to utilize artificial neural network (ANN) architecture. In recent years, optical interference units (OIUs) [13], photonic synapses implemented with phase change materials (PCMs) [14], and deep diffractive neural networks (D^2^NN) [15] have been exploited as three major technical trends towards optical AI accelerators. Multiply–accumulate (MAC) operations, the major computation load in the feedforward neural network, can be implemented by light–matter interactions at the speed of light propagation. Optics is found to be potentially capable of supporting next-generation computing [16,17]. In addition, ANNs have excellent scaling and speed advantages if complex logic operations and calculations could be performed end-to-end and highly parallel multi-input–multi-output to avoid the serial cascading of logic gates. Qian et al. proposed a logic computing architecture based on D^2^NN [18]. Spatial diffractive masks were put into series to form the computing unit. Seven typical logic bitwise operations were realized. However, it is still challenging to perform logic operations with large bit-width on photonic integrated chips (PICs) considering the light propagation loss and nonlinearity difficulty [19]. Bitwise optical logic unit is unnecessary as D^2^NN optical computing does not need to follow the route of Boolean algebra in the electronic design. D^2^NN based on modulated wavefront and optical diffraction shows the excellent capability of large-scale optical fully connected (FC) neural networks. Thus, we need to thoroughly understand the potential of D^2^NN and optical FC neural networks and construct a general process for designing and training such networks.

This paper presents a generalized strategy for realizing optical logic full-adder, which fully exploits the parallelism of the on-chip D^2^NNs. The Tanh function is implemented as the activation function, which can be realized by quantum dots and integrated onto silicon chips. A 4-bit D^2^NN logic full-adder is designed, and a 100% accuracy inference is demonstrated under complete dataset simulation.

## 2. Architecture of the D^2^NN Optical Full Adder

**Figure 1:**
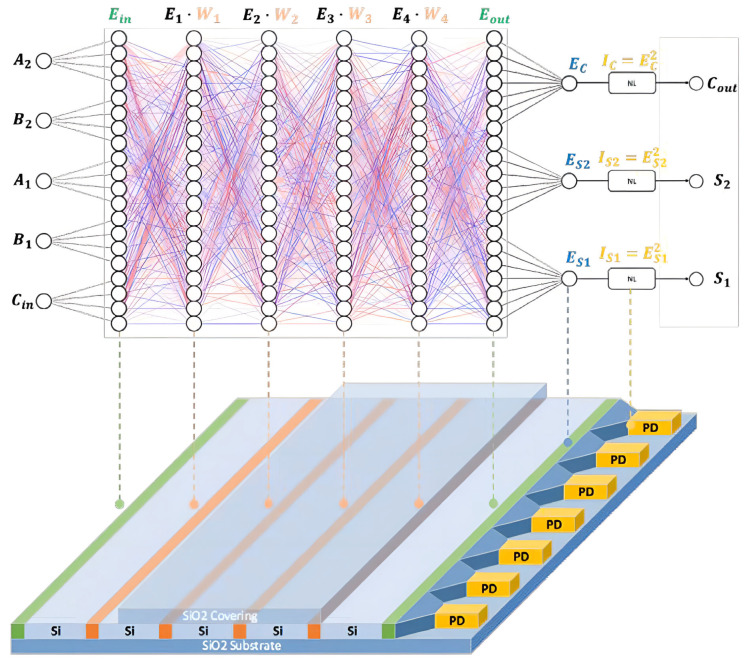
The architecture of the D^2^NN full adder allows the signal to propagate from left to right in parallel through the computing network and activation layers. The output ports outlet the logic adding results.

D^2^NN can be realized through the lightwave propagation and diffraction process. As shown in Figure 1, the proposed on-chip D^2^NN full adder is featured with a metasurface composed of the sub-wavelength size of dielectric structure array. Each layer of the neural network (meta line) is composed of multiple slots (meta-atom) filled with silicon dioxide. The slot’s effective refractive index value is between that of silicon dioxide (n = 1.5) and silicon (n = 3.4), and it confines the lightwave propagation pathway. The lightwave propagates along the slot and is diffracted after leaving the slot. During this process, the phase can be modulated from 0 to 2π by varying the slot length by a few micrometers, and the transmission efficiency exceeds 94% [20]. The modulated lightwave propagates towards the following meta line and realizes a full connection between these two columns of the slots. The phase shift caused by the meta-atom represents the weight connecting the two layers, and this weight is modulated by the feature size of the slots. One or several slots work as a neuron, of which the transmission coefficient of the *l*th slot is $T^{l}=t^{l}exp\left(j\emptyset^{l}\right)$, where $t^{l}$ represents the amplitude modulation of the *l*th diffraction slot on the input light field (due to the high energy transfer efficiency, this coefficient is very close to 1), and $\emptyset^{l}$ represents the phase modulation of the *l*th slot, which is adjusted by slot length.

Then, we analyze the mathematical expression of the full connections between layers. If we denote the lightwave coming into the mth metaline at nth slot as $A\left(n\right)$, after propagating through the slot, the lightwave becomes $A\left(n\right)T\left(n\right)$, and then diffraction happens when the lightwave leaves the meta-atom. The angular spectrum method is used to analyze the diffraction process. The transition function between meta-atoms (*n*th slot in *m*th metaline with *r*th slot in (*m* + 1)th metaline) is fixed and can be represented as *U*(*n*, *r*). Thus, the electric field of light before entering the (*m* + 1)th metaline at *r*th slot becomes $\sum_{n=1}^{n=N}A\left(n\right)T^{m}\left(n\right)U\left(n,r\right)$. Thereafter, full connections (multiply and accumulate operation) between neurons can be realized through the on-chip diffractive neural network.

**Figure 2:**
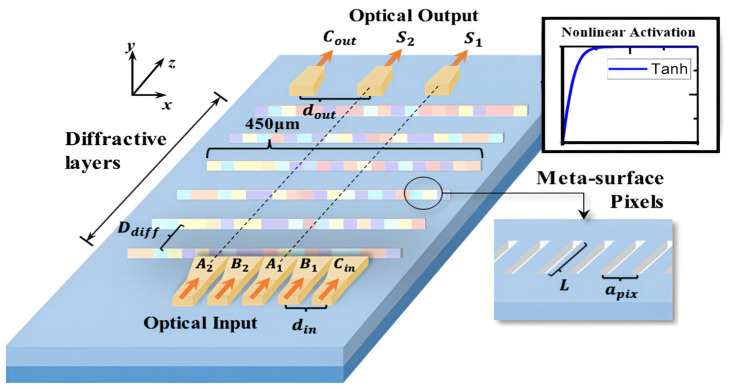
Schematic of D^2^NN 2-bit full adder.

The schematic diagram of the proposed on-chip diffractive neural network, using a 2-bit full adder as an example, is shown in Figure 2. The D^2^NN is designed on a 220 nm SOI substrate. Each layer is a trained phase mask with fully etched hollows filled with SiO_2_, forming 150 nm wide metasurface pixels. The distance between the centers of two neighboring pixels (a_pix_) is 500 nm. The phase delay can be controlled linearly by the propagation length L [20]. The physical implementation of the nonlinear activation is achieved by filling PbS quantum dots into specific diffraction layers [21,22,23,24,25,26,27]. The distance between each layer (D_diff_) is 60 μm. Logic inputs, interleaved with two operands, A and B, are placed along the x-axis with an input port distance (d_in_) of 12 μm. In this way, maximum interference between neighboring logic bits is achieved. Output ports are aligned with the inputs, with an output port distance of 24 μm. Tapers of 11 μm to 450 nm are also placed at the input and output ports to provide sufficient coupling to and from the diffraction area. The outputs are connected to photodetectors (PDs) for readout and nonlinear activation.

Logic operations can be regarded as an end-to-end transformation between input bits and output bits. For example, for 1-bit full adders, the input elements are two 1-bit operands *A*, *B* and carry-out bit *C_in_*_,_ while the output elements are the 1-bit sum *S* and the carryout bit *C_out_*. The following equations describe the mathematical expression between inputs and outputs, where ⊕ represents the XOR operation.

(1) $$S=A⊕B⊕C_{in}$$

(2) $$C_{out}=AB+C_{in}\cdot \left(A+B\right)$$

**Figure 3:**
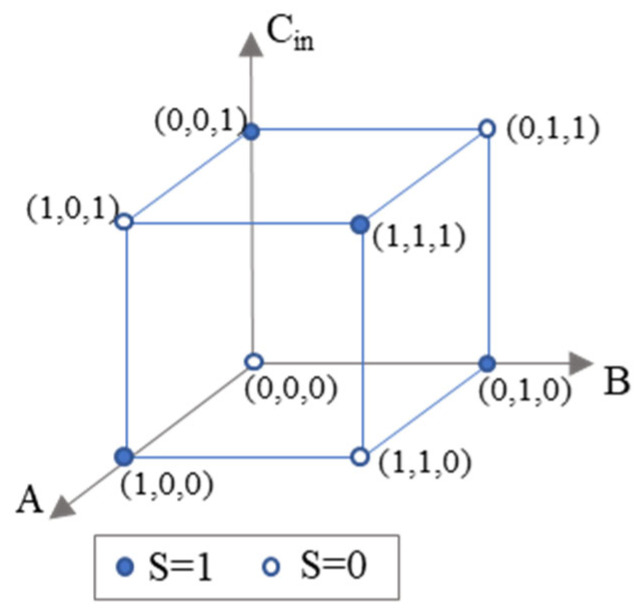
The task of realizing 1-bit full adder equals to a classification problem in Descartes coordinate system.

This task can be regarded as a classification problem, as shown in Figure 3. Here, the three axes indicate the three inputs, and the color of the vertices on the cube represents the value of *S*. For algebraic derivation, the problem lies in solving matrix equations. However, such equations cannot be solved with a linear matrix, as we cannot divide the space in Figure 3 with a plane to have the solid circles (*S* = 1) and the hollow circles (*S* = 0) separated. Thus, we need specific design methods to implement full adders using D^2^NN.

**Figure 4:**
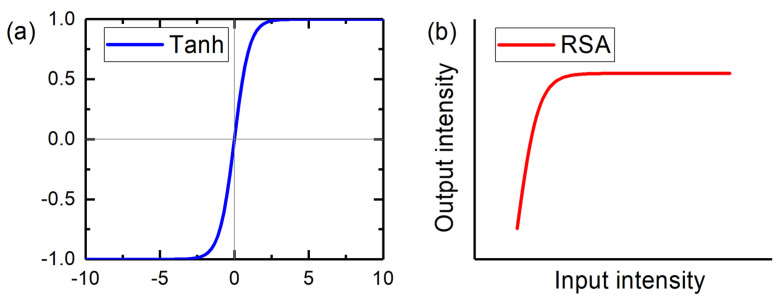
(**a**) Tanh activation function and (**b**) RSA nonlinear activator.

In deep neural networks, a linear mathematical sum operation of weighted inputs and a nonlinear mathematical activation operation of the sum are combined to mimic neurons. The sum of weighted inputs is realized during the process of propagation and diffraction. Mathematical nonlinear activation functions have been developed with simple descriptions such as sigmoid, tanh, rectified linear unit (ReLU), leaky ReLU, max-out, and exponential linear unit (ELU), among others. Optical elements have been investigated to realize these nonlinear functions, such as the Mach–Zehnder interferometer with optical–electrical conversion feedback [28,29] to mimic ReLu and micro-ring resonator [30] to realize sigmoid and ReLU. Specific nonlinear optical materials have also been developed to fulfill the nonlinear computing requirement. The reverse saturable absorption (RSA) phenomenon in low-dimensional materials shows a perfect compatibility with the activation curve of Tanh. Based on nonlinear optical theory, the absorption coefficient *α* of the medium is expressed as $\alpha =\frac{\alpha_{0}}{1+I/I_{sat}}+\beta I$, consisting of a linear absorption coefficient *α*_0_, a nonlinear absorption coefficient *β*, the input intensity *I*, and saturation intensity *I*_sat_. For the RSA nonlinearity, the relationship between transmittance *T* and input light intensity *I* is simplified as $T\left(I\right)=1-T_{ns}-∆T\times [-exp(-\beta I)]$ [31,32], where Δ*T* is the modulation depth and *T*_ns_ is the non-saturated loss. T(I) can be solved by this formula for a given input intensity *I*, and the output light intensity can be obtained by $I_{out}=I\times T(I)$ [33]. Subsequently, the relationship curve between *I*_in_ and *I*_out_ as the nonlinear activator is similar to the positive part of the Tanh function [32], as shown in Figure 4.

In this work, we utilize the Tanh function as the activation function. The primary consideration is that this function can be realized by quantum dots, which can be integrated into silicon chips. Lead sulfide (PbS) quantum dots (QDs) exhibit strong quantum confinement arising from large exciton Bohr radius (20 nm) and large third-order nonlinear susceptibility [21,22,23]. For a given size, the nonlinearities of PbS nanocrystals will be much larger than that of other semiconductor QDs such as GaAs and CdS, where the χ^(3)^ value was reported to be about 1.0 × 10^−8^ esu for PbS QDs in polymer matrices [24]. The impressive third-order nonlinearities make PbS QDs have great potential to achieve excellent RSA performance. Their ultrafast carrier excitation and long exciton lifetime imply an ultrafast saturation response and low saturation intensity. The long exciton lifetime (~µs) for colloidal PbS QDs emitting in the near-infrared region is attributed to the decreased radiative recombination rate due to both low transition energy and a strong dielectric screening effect [25]. Consequently, PbS QDs are expected to achieve RSA performance with low saturation intensity as a nonlinear activation function in ONN.

It has been theoretically proven that our D^2^NN structure has the capability to realize arbitrary logic computing units. First, Kulce et al. proved that diffractive layers can perform arbitrary complex matrix operations as long as the adjustable neurons of the diffractive network are large enough [20], indicating that the combination of diffractive layers and nonlinear layers is mathematically equivalent to the standard multilayer perceptron (MLP) with complex values. Second, Hornik et al. proved that even the simplest three-layer perceptron with only one single hidden layer can approximate the arbitrary continuous function with any precision requirements [28]. As all logic operations can be regarded as a continuous function that exactly fits the truth table, our optical diffractive architecture, which has a mathematical form similar to that of a multilayer perceptron (MLP), can implement arbitrary logic operations. Last but not least, Du et al. proved that the MSE loss of an MLP can drop to 0 at an exponential rate using the simple gradient descent (GD) method as long as network parameters are large enough [29]. This result implies that training arbitrary logic operations using our architecture can yield an ideal result in finite time, even when we use standard optimization methods like GD.

## 3. Results

### 3.1. A Demonstration of the Fundamental XOR Function

**Figure 5:**
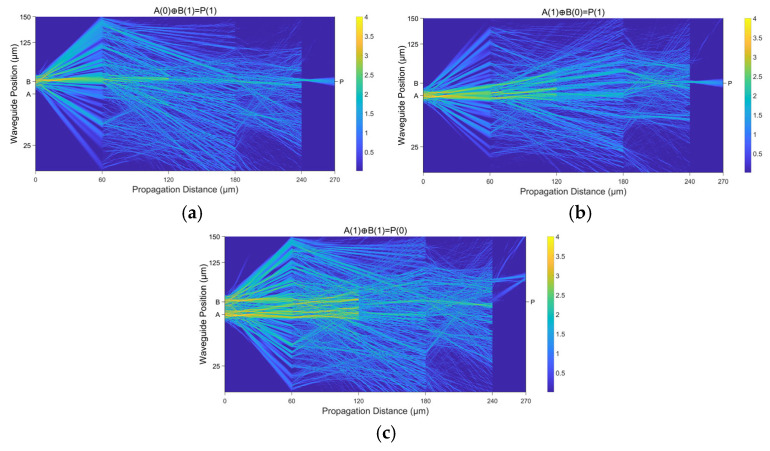
The demonstration of the fundamental XOR function realized by the D^2^NN structure with three different input cases. (**a**) A(0)⊕B(1) = P(1); (**b**) A(1)⊕B(0) = P(1); (**c**) A(1)⊕B(1) = P(0).

In this study, we demonstrate the successful implementation of a logical XOR operation using a diffractive neural network architecture comprising four diffractive layers. After the second layer, a PdS layer is introduced, which introduces nonlinear saturation effects critical for error truncation and signal modulation. The optical field propagation and intensity distribution across the multilayer system are visualized in Figure 5, illustrating the dynamic interplay of light diffusion through diffraction and coherent interference-mediated wavefront shaping. The input coherent light is a Gaussian beam at a wavelength of 1.55 μm. It undergoes spatial spreading via diffraction, followed by weighted summation through constructive and destructive interference at each layer, enabling precise control over the propagation direction and phase amplitude profiles. The PdS nonlinearity, acting as a thresholding mechanism, effectively truncates redundant signals and suppresses error accumulation by saturating undesired high-intensity components. This dual mechanism—diffractive spatial modulation and nonlinear amplitude filtering—facilitates the progressive transformation of input optical patterns into the XOR logic output. Experimental validation confirms that the proposed architecture achieves 100% accuracy in XOR classification, which proves the efficacy of the proposed network in solving linearly inseparable problems. The results highlight the synergy between wave-based physical computation and nonlinear material responses for advanced optical computing paradigms.

### 3.2. Training of the 4-Bit Full Adder

In this study, we demonstrate the successful implementation of a 4-bit full adder using a diffractive neural network architecture. There are sixteen linear diffractive layers and two nonlinear layers, which are located after the fourth and twelfth linear layers, respectively. The weight of neuron connections $T\left(n\right)$ can be modulated by setting a proper size of meta-atom (width, length). The modulation coefficients are obtained through training. The Adam optimizer is used to perform the gradient descent and error backpropagation of Fresnel diffraction. Similarly to a deep neural network, D^2^NN takes forward propagation and gradient update (backpropagation) to train the parameters. The system’s performance is highly determined by the training method and training quality. Please note that the forward propagation in D^2^NN has two distinct points compared to a general deep fully connected neural network: Firstly, the weights are complex numbers which are controlled by the slot waveguide. Secondly, the lightwave leaving a slot waveguide is diffractively distributed to the next stage of slots. The incident light field in layer *i* is denoted as $E_{i}\left(x\right)$.

(3) $$E_{i+1}\left(x\right)=\frac{1}{j\lambda }\int_{-\infty }^{\infty }E_{i}\left(x_{0}\right)\frac{e^{jkr}}{r}K\left(\theta \right)dx_{0}$$

(4) $$r=\sqrt{{\left(x-x_{0}\right)}^{2}+d^{2}}$$

where $K\left(\theta \right)$ is the inclination factor, θ is the angle between the diffraction direction and normal direction of the layer, and *d* is the distance between layers.

The diffraction propagation is a linear process. The forward propagation can be represented by a cascaded product of transport matrix and diffractive weight matrix, and the output light field $E_{out}\left(x\right)$ can be calculated thereafter. The discrepancy between the real output $E_{out}\left(x\right)$ and ideal output (ground truth) can be represented by the loss function, of which mean square error (MSE) is one of the most commonly used cases.

(5) $$e_{MSE}=\frac{1}{m}\sum_{i=1}^{m}{\left(E_{i}-{\hat{E}}_{i}\right)}^{2}$$

where $E_{i}$ and ${\hat{E}}_{i}$ denote the real output and targeted output, respectively. *m* is the total number of neurons in the output layer. The loss function is critical to the network training, as it determines the gradients and parameters that are updated. MSE exhibits good performance in maintaining the Gaussian-like field. The accuracy of all input cases reaches 100% after 1000 epochs, which proves the feasibility of implementing the 4-bit full adder using the mentioned structure.

### 3.3. Realization of the 4-Bit Full Adder

**Figure 6:**
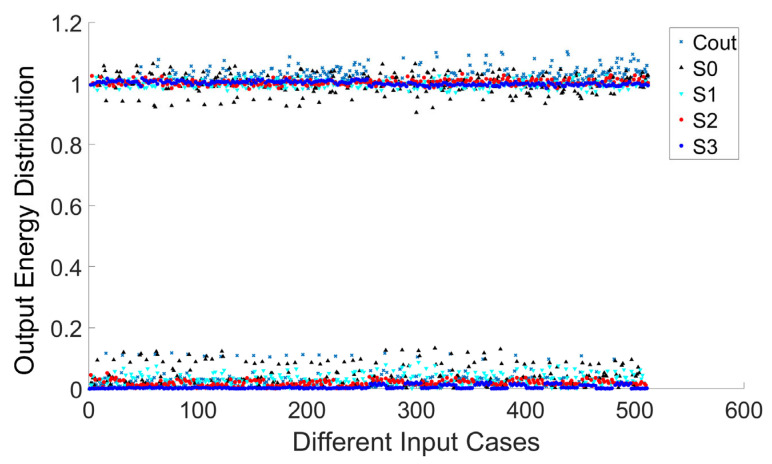
Energy distribution of the output bits of the 4-bit full adder.

**Figure 7:**
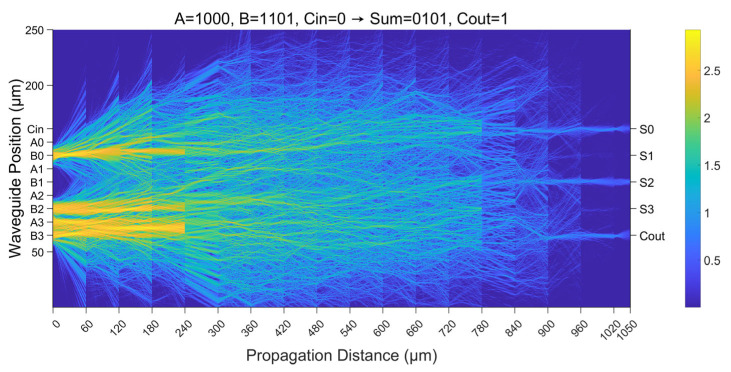
Simulated results of the 4-bit full adder using the proposed D^2^NN.

Figure 6 shows the amplitude of output bits of all 512 input cases of a 4-bit full adder. The accuracy of the full adder achieves 100% for all input scenarios, and our design can distinguish logic 1 from logic 0 very well in the worst output cases. Furthermore, we present the optical field propagation and intensity distribution in Figure 7. This illustrates one of the input cases, where the operands are 1000 and 1101, while the carry-in C_in_ is 0. As expected, the output of summation is 0101, and the carry-out C_out_ is 1.

## 4. Conclusions

In this work, we propose a novel architecture for a 4-bit optical full adder based on a diffractive neural network. The physical parameters of each diffractive layer are well adjusted to perform a proper linear transformation of the light field, and a nonlinear optical process is used to increase the expressiveness of the optical network. An end-to-end 4-bit optical logic adder is implemented with 100% accuracy. For future work, optical adders with a larger bit width can be realized by cascading several adders. This lays an essential foundation for achieving general-purpose optical computing.

## Data Availability

The datasets generated and/or analyzed during the current study are available from the corresponding author (J.Z.) on reasonable request.
